# Phenol–Furfural Resin/Graphite/Ag-Based Electrically Conductive Adhesive Composites from Waste Bagasse with Enhanced Thermo-Electric Properties

**DOI:** 10.3390/polym15153283

**Published:** 2023-08-03

**Authors:** Syeda Mahnoor Zehra, Maryam Bibi, Azhar Mahmood, Abraiz Khattak, Muhammad Zeeshan Asad, Syeda Hijab Zehra

**Affiliations:** 1 School of Natural Sciences, National University of Sciences and Technology, H-12, Islamabad 44000, Pakistan; syedamahnoor1111@gmail.com (S.M.Z.);; 2 US Pakistan Center for Advanced Studies in Energy, National University of Sciences and Technology, H-12, Islamabad 44000, Pakistan; 3 Department of Chemistry, Loughborough University, Loughborough LE11 3TU, UK; zeeshanasad062@gmail.com; 4 Department of Earth and Environmental Science, Bahira University, Islamabad Campus, H-11, Islamabad 44000, Pakistan

**Keywords:** silver, green material, biomass, furfural, PFR, conductivity

## Abstract

This study describes the preparation and evaluation of phenol–furfural resin (PFR) from bagasse and its nanocomposites for electrically conductive adhesive (ECA) application. PFR was prepared with furfural extracted from bagasse using a modified acid digestion method. Three different formulations of PFR nanocomposites with conductive nanoparticles, i.e., PFR-silver, PFR-graphite, and PFR-silver + graphite, were prepared using 20, 40, and 60 *w*/*w*% of fillers via the impregnation method. The resultant products were characterized using FT-IR, SEM, EDS, and XRD spectroscopy. Electrical conductivity was measured using a four-probe technique, while band gap was calculated via Tauc plots. The results exhibited a significant rise in electrical conductivity of insulating virgin PFR from 2.6 × 10^−4^ Scm^−1^ to 8.2 × 10^−1^ Scm^−1^ with a 40 and 20 *w*/*w*% blend of Ag and graphite in PFR. This synergism was exhibited because graphite and Ag NPs supply excellent junctions for building networks. Both tend to coalesce due to van der Waals forces and high surface energies. Therefore, conductive pathway numbers can be increased, and the contact area can be effectively enlarged. This ternary composite exhibited the lowest bandgap energy value, i.e., 3.1 eV. Thermogravimetric temperature values T_0_ and T_deg_ were increased up to 120 °C and 484 °C, respectively, showing a significant increase in thermal stability. Therefore, the resultant nanocomposite material has good potential to be employed as an ECA in the electronic industry.

## 1. Introduction

The current world economy is affected by the scarcity of basic human necessities like clothing, shelter, domestic fuel, and food due to feedstock reserve depletion. Experts have been investigating different options for exploring alternate means for achieving better key solutions for such basic needs. To reduce the world’s dependency on fossil fuels, the implementation of alternative sources such as fuels from renewable resources, production of green materials, and chemicals is now gaining strength [1,2,3,4].

With the advancement of science and technology, there is a need for the development of electronic units that are portable and very small in addition to having highly integrated functions [5]. Consequently, the electronic industry demands new interconnection bonding materials that can offer good electrical conductivity, satisfactory thermal stability, and flexibility, along with low cost and environmental friendliness. Polymers produced from renewable resources are also of great interest due to their lower cost and high abundance [6,7,8]. Applications of furfural have been found in novolac-type green adhesive phenol furfural resin because of the presence of aldehyde group functionality, as well as the fact that it can be extracted using renewable non-edible/edible feedstock. Furfural can be incorporated into phenolic resin using furanylmethylol and furanylmethine groups [9,10]. Using phenolic linear resins of resorcinol formaldehyde, resorcinol formaldehyde urea branched resins, and resorcinol furfural phenol resins, A. Pizzi et al. investigated its structures. They reported that various resorcinol percentages were required for equal resin performance as adhesives. The performance of resin was greatly improved through increasing the proportion of oligomers having methylol groups or oligomers containing resorcinol. However, in such resins, higher furfural molecular weight is the determining parameter compared to formaldehyde [11]. In another study, resorcinol–phenol–furfural, resorcinol–furfural, and phenol–resorcinol–furfural resins of cold settings were synthesized for substituting cold-setting formaldehyde-based resin. Such phenolic adhesive resins have the benefit of less volumetric shrinkage upon curing [12]. Electrically conductive adhesives (ECAs) are excellent options to be used as an interconnection material in the electronic industry since they offer improved electrical performance, fine pitch interconnect, environmental friendliness, and low processing temperatures [13]. ECAs are composed of two main parts: one is polymeric and the other is filler. The polymeric part may consist of polymeric resins such as polyurethane, polyimide, silicones, acrylates, epoxy, phenolic resins, etc. [14], while the conductivity will be provided using conductive fillers such as Ag, Ni, graphite, graphene, CNTs, etc. [15,16,17]. Various conductive fillers have been studied, including zinc complexes [18,19], metal oxide [20], cadmium [21], cobalt nanoparticles [22], etc. However, some issues like poor interconnection between the polymer resin and conductive fillers and aggregation can be solved through optimizing the quantities and percentages of conducting fillers and resin. Suherman et al. reported a conductivity of about 28 Scm^−1^ through using about 80% of graphite along with epoxy resin, resulting in improved mechanical and electrical performances of epoxy–graphite composites [23]. The research group of Duarte and Paulo [16] studied epoxy resin composites consisting of single- and multiwalled carbon nanotubes (SWCNTs and MWCNTs) and calculated a volume resistivity of about 1 × 10^1^ and 1 × 10^6^ Ωcm for SWCNTs and MWCNTs. In another work, Dhakate et al. [24] used graphite (natural and synthetic) as the conductive material with epoxy resin for bipolar plates, with the highest conductivity at about 150 Scm^−1^. Nayak and colleagues [25] extensively reviewed adhesive resins with various conductive fillers and reported a thermal conductivity of 0.45 W/mK. and 10^1^ Scm^−1^ as the electrical conductivity for epoxy/single-walled nanotubes of carbon-based ECAs. Tang et al. [26] designed and synthesized silicone resin with functional silane and applied this as silver conducting ink, where Ag-silicone resin with 53.93 wt.% Ag showed the lowest resistivity of 1.43 × 10^−6^ Ωm and was then applied as the conductive silver ink. Qian et al. presented ECAs with higher conductivity and a lower content of conducting filler through utilizing micro- and nano-scale fillers’ synergistic effects. They investigated this using silver microns coated on copper microns (Ag@Cus, Ag@NWs) as ECAs. In the binary ECA compound, the bulk resistivity achieved was 9.42 × 10^−5^ Ωcm with a 1:9 mass ratio and filler content of only about 60 wt% [27]. Ailing et al. discovered ECA composites using MXene Ti_3_C_2_Tx nanosheets through simple blending with epoxy resin. Different properties like electrical conductivity, mechanical strength, and both tensile and impact strength were about 4.52 × 10^−4^ S/cm, 66.2 MPa, and 24.2 kJ/m^2^, respectively [28]. The mechanical and resistive properties of Cu-modified nano-Ag nanoparticles were studied by Zhang et al. for applications in electronic industries such as in integrated circuit packaging or LED packaging to replace traditional solders and Pb-free solders. They explored electrical resistivity and the shear strength of the prepared Ag-modified nanoparticles of Cu in terms of filler content, curing time, and curing temperature. Finally, the best composites have a resistivity of 5.159 × 10^−6^ Ωcm and 6.121 MPa of mechanical strength, with about 170 °C curing temperature in 100 min with 23.4 vol% of filler content [29]. Giorgio et al. prepared conductive composites for scientific applications and 3D printing. It is well known that several particles like metal powder, composite polymers, and conductive fillers are preferred for ECAs, but they synthesized novel conductive resin using Poly (ethylene glycol) diacrylate for cross-linking. Poly(3,4-ethylenedioxythiophene) was utilized as filler, which then exhibited 0.05 S/cm of electrical conductivity to be applied in stereolithography [8]. Zhang Rongwei and his co-workers reported various resin ratios with Ag-coated flakes prepared through a simple chemical reduction method. The lowest bulk resistivity was shown with only one composite at 80 wt% of flakes, which results in 1.3 × 10^−3^ Ωcm, which has the potential to be used as conductive ink [17].

In the present work, the authors report the preparation of ECAs through the incorporation of nano-conductive fillers (graphite and Ag) into a matrix of PFR. The resultant nanocomposites were investigated using several characterization techniques including SEM/EDS, FTIR, and XRD, while thermal and electrical properties were investigated as well. The main aim of this research is to synthesize bio-based composites involving feasible and cost-effective methods for its synthesis. Therefore, such conductive resin made from renewable biomass has the potential to be used as a conductive adhesive (ECA), which can be then utilized on an industrial level in parts of electronic equipment as well as electrically conductive inks. To the best of our understanding, these Ag/graphite composites embedded in PFR have not been explored yet [30].

## 2. Materials and Methods

### 2.1. Materials

Sugarcane plant was purchased from the local market of the city of Wah Cantt, Pakistan. It was first washed and then allowed to pass through a sugarcane presser to obtain bagasse. Subsequently, this bagasse was shade-dried for about 14 days followed by grinding and drying in an oven at about 50 °C for half an hour to ensure it was moisture free. Chloroform (99%), AlCl_3_·6H_2_O (99%), HCl (37%), NaCl (99%), KOH (99%), Phenol (99%), Urea (99%), Hydrazinium chloride (99%), Silver Nitrate (99%), and Graphite (dry battery cells) were used. All chemicals were procured from Daejung, Korea, while deionized water was used for all solution preparations.

### 2.2. Furfural Extraction from Bagasse

A mixture of catalysts comprising equal quantities of NaCl and AlCl_3_.6H_2_O was prepared. Next, 12% HCl solution along with 2 *w*/*v*% of the catalyst material was used as an extraction medium. Bagasse was charged in 1:10 proportion of extraction medium; it was then mixed and agitated to ensure proper mixing of solution and powder. Subsequently, the prepared material was poured into a 1000 mL round-bottom flask attached with a condenser and heated at 100 °C for 2.5 h with magnetic stirring in an electrothermal heating mantle. After bagasse digestion, the distillate was transferred into a separating flask. Chloroform was added into the distillate and shaken well to extract furfural into the chloroform phase. Evaporation of chloroform, via rotary evaporator yielded 53% furfural.

### 2.3. Synthesis of Phenol-Furfural Resin

Phenol furfural resin was prepared according to the method reported by Zeeshan et al. [31]. Furfural and Phenol were reacted in a molar ratio of 0.9:1. About 12.5 g of phenol and 0.25 g of KOH as catalyst were taken into a 100 mL three-necked round-bottom flask, and both were melted at 45 °C until KOH was dissolved. Subsequently, furfural (11.5 g) was dropwise added in about 30 min into flask with constant stirring. Now, the temperature of the system was raised up to 135 °C for about 2 h, and a liquid product having dark colour was obtained. In order to cease the polymerization process, and to remove unreacted phenol, the product was placed in a vacuum oven for 24 h. This results in the formation of PFR having no phenolic content. Moreover, when the resin was heated for more than 3 h, a solid dark-colour resin was achieved without phenol, which was used for the studies of SEM analysis.

### 2.4. Graphite Powder

The graphite rod of an AAA battery cell, having a weight of about 3 g and diameter and height of 0.41 × 1.75 mm was dismantled and ground, followed by sieving using a 0.3–0.4 nm pore-sized sieve. This fine powder was used for the preparation of graphite conductive composite synthesis.

### 2.5. Synthesis of Silver Nanoparticles

A simple approach of chemical reduction method [32] was used for the Ag NP synthesis. Sodium hydroxide was used as an accelerator while hydrazinium chloride was used as the reducing agent. In the first step, 0.68 g of AgNO_3_ was dissolved in 40 mL of DI water and kept at 90 °C for 5 min with vigorous stirring. Aqueous solutions of sodium hydroxide (0.64 g/10 mL DI water) and hydrazinium chloride (0.45 g/10 mL DI water) were prepared and simultaneously added into silver nitrate solution, where strong effervescence was noticed. This reaction was allowed to continue for about 1 h until the fizziness disappeared. The obtained precipitates were washed with DI water 5 times at a speed of 5000 rpm to remove any kind of unreacted material and were dried in a vacuum oven at 60 °C for 24 h to obtain greyish silver nanoparticles.

The graphite rod of a 3A battery cell was dismantled and ground, followed by sieving using a nanometre-pore-sized sieve. This fine powder was used for the conductive nanocomposite synthesis.

### 2.6. Preparation of Composites and Nanocomposites

#### 2.6.1. Preparation of Graphite–Resin Composites

Graphite–resin composites were prepared in 20:60, 40:40, and 60:20 *w*/*w* ratios of graphite and PFR resin with the sample codes G-20, G-40, and G-60, respectively. For the impregnation of graphite particles, a mechanical stirring process was performed at 25 °C for 25 min. Afterward, samples were placed in a vacuum oven at 90 °C for 24 h for complete drying. When the concentration of graphite powder was increased above 60 *w*/*w* ratio, saturation of particles was observed, and mechanical stirring became difficult. This results in nonhomogeneous composite formation along with weak interaction between particles and polymers.

#### 2.6.2. Preparation of Ag-Resin Nanocomposite

The silver and resin nanocomposites were prepared with the same procedure used for the graphite resin composite preparation. Samples were labelled as S-20, S-40, and S-60 for the compositions as 20:60, 40:40, and 60:20 *w*/*w* ratios of Ag and PFR resin.

#### 2.6.3. Preparation of Graphite-Ag-Resin Nanocomposite

For ternary composite preparation, both graphite and Ag nanoparticles were used as per the aforementioned method. These are coded as SG-20, SG-30, and SG-40 for 20:40:40, 30:30:40, and 40:30:40 *w*/*w* ratios of silver, graphite, and PFR resin, respectively.

## 3. Analysis Techniques

A Perkin Elmer Spectrum 100 FT-IR Spectrometer (Los Angeles, CA, USA) was used in the frequency range of 350–4000 cm^−1^ to study the characteristic peaks of furfural and resin. A KBr disc was used as sample holder material. A BRUKER D8 advanced X-ray diffractometer (Moscow, Russia) equipped with Cu K alpha 0.154 nm wavelength radiation sources was employed in theta range of 10 to 80° to examine the crystallinity of the filler materials. The surface topography of the nanocomposites was examined using a JSM-6490A, JEOL (Shimadzu, Kyoto, Japan) SEM instrument, while images were captured at resolution scale of 10 µm and 1 µm. Thermogravimetric analysis (TGA) was conducted via an SDT 650 TA instrument (Los Angeles, CA, USA) under inert conditions. Initial temperature was 26 °C and was increased to 900 °C with a 10 °C/min ramp rate in an inert nitrogen atmosphere. Jasco V-770 via UV-Visible Spectrophotometer (Shimadzu, Kyoto, Japan) was used for the bandgap measurements via the diffuse reflectance spectroscopic technique (DRS). Conductivity of the samples was measured using a four-point probe method via JANDEL RM3000 (Loughborough, United Kingdom) test unit. The technique was used to measure the resistivity of the samples from which the conductivity was calculated through taking the reciprocal of resistivity. A DC supply with constant current was used for all the measurements.

## 4. Results and Discussion

### 4.1. Fourier Transform Infrared (FTIR) Spectroscopy

**Figure 1:**
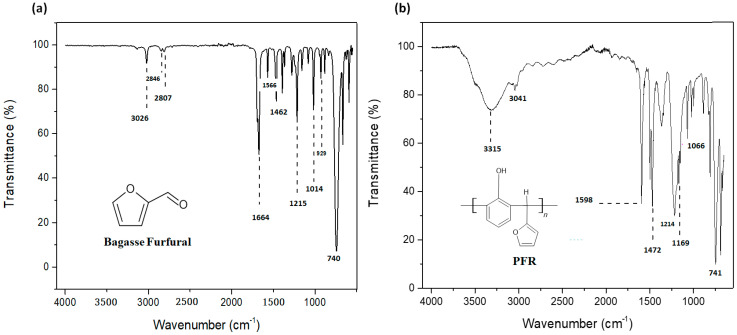
FT-IR spectrum of (**a**) Bagasse Furfural (**b**) PFR.

**Figure 2:**
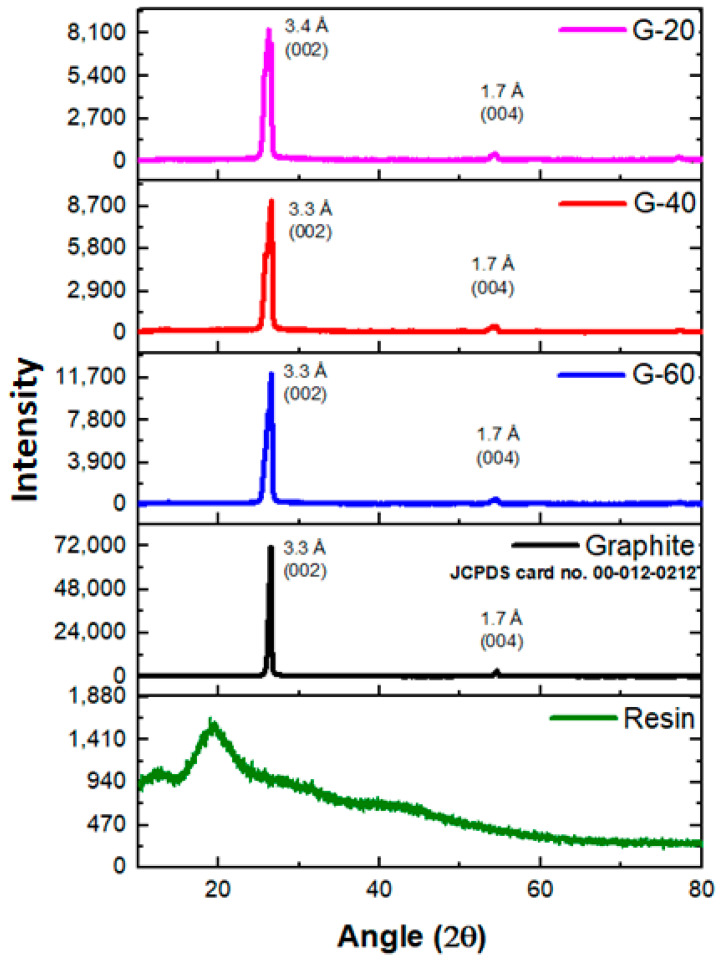
XRD pattern of resin, pure graphite, and graphite composites.

In order to identify furfural, FTIR studies were carried out. It was confirmed by the band at 3026 cm^−1^ in the FTIR spectrum, which is due to the Ar-H stretch, as shown in Figure 1. The aldehyde group was confirmed by the presence of two symmetric and asymmetric (H-C=O) bond stretches at 2846 cm^−1^ and 2807 cm^−1^ [31]. The intense band at 1664 cm^−1^ is due to a C=O stretch. Moreover, the two bands at 1566 cm^−1^ and 1462 cm^−1^ are corroborated by the presence of an aromatic ring C=C bond. The presence of a furan ring was ensured by a 1215 cm^−1^ bond stretch, while the 1014 cm^−1^ band stretch reflects the presence of a C-H plane. A=C-H out-of-plane bending vibration and C-H out-of-plane (monosubstituted) bending vibration are exhibited at 929 cm^−1^ and 740 cm^−1^, respectively [31]. The resin is corroborated by an absorption band at 3315 cm^−1^ due to -OH (Figure 2). The polymer containing furfural and phenol monomers exhibits aromaticity due to which stretching bands of Ar C-C, Ar-OH, and Ar-H appeared at 1598 cm^−1^, 3315 cm^−1^, and 3041 cm^−1^, respectively. Bands at 741 cm^−1^ and 1472 cm^−1^ were of monosubstituted C-H out of plane and C=C present in the furan ring. A band appeared at 1214 cm^−1^ due to C-O moiety. C-O-C asymmetrical stretching vibrations resulted in the 1169 cm^−1^ and 1066 cm^−1^ bands. All these bands have confirmed the successful synthesis of phenol furfural resin.

### 4.2. X-ray Diffractometry (XRD)

In order to investigate material properties like diffraction planes, interlayer spacing, and crystallinity, XRD studies were carried out. The XRD results of PFR resin, graphite powder, and their composites were shown in Figure 2. The phenol furfural resin has depicted no sharp peak, which confirms its amorphous nature. A similar result was reported by L Guo et al. [33]. On the other hand, sharp and intense peaks were shown by graphite and all of its composites. These peaks perfectly match JCPDS card no. 00-012-0212. The intense characteristic diffraction pattern of pure graphite has exhibited two diffraction peaks: one at a 2θ value of 26.7° with interlaying spacing of 3.3 Å and (002) plane and the other at a 2θ value of 54.5° having d-spacing 1.7 Å and (004) diffraction plane. The XRD results in Figure 2 depict a detailed description of interlayer spacing and diffraction planes of graphite nanocomposites. The interlayer spacing of composites remained the same, i.e., 3.3 Å and 1.7 Å for the two peaks, respectively. This confirmed that the graphite particles remain unchanged even when mixed with the phenolic resin, and no reaction has occurred between the graphitic sheets. This is proved by the absence of any peak that suggests the existence of graphite oxide, graphene oxide, etc.

**Figure 3:**
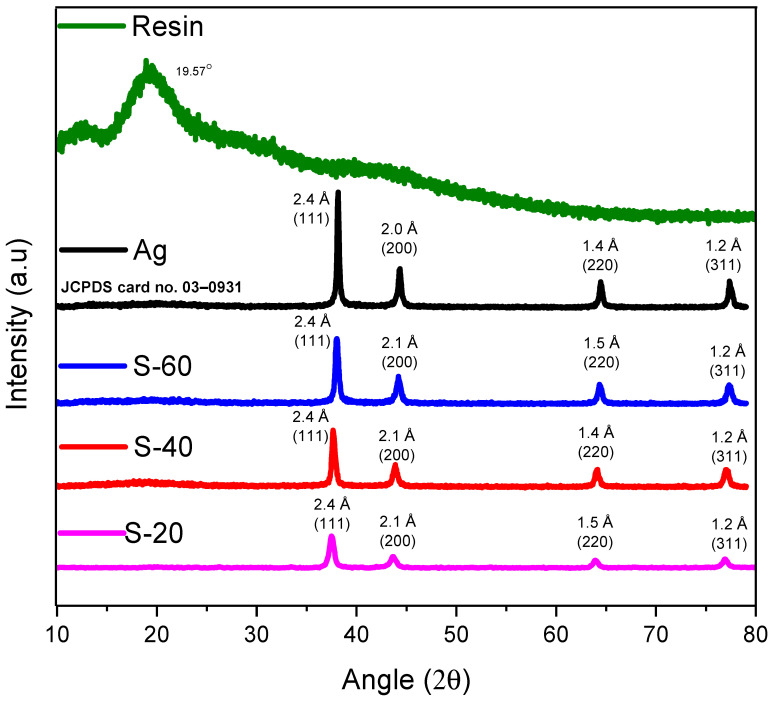
XRD pattern of Ag and Ag composites.

**Figure 4:**
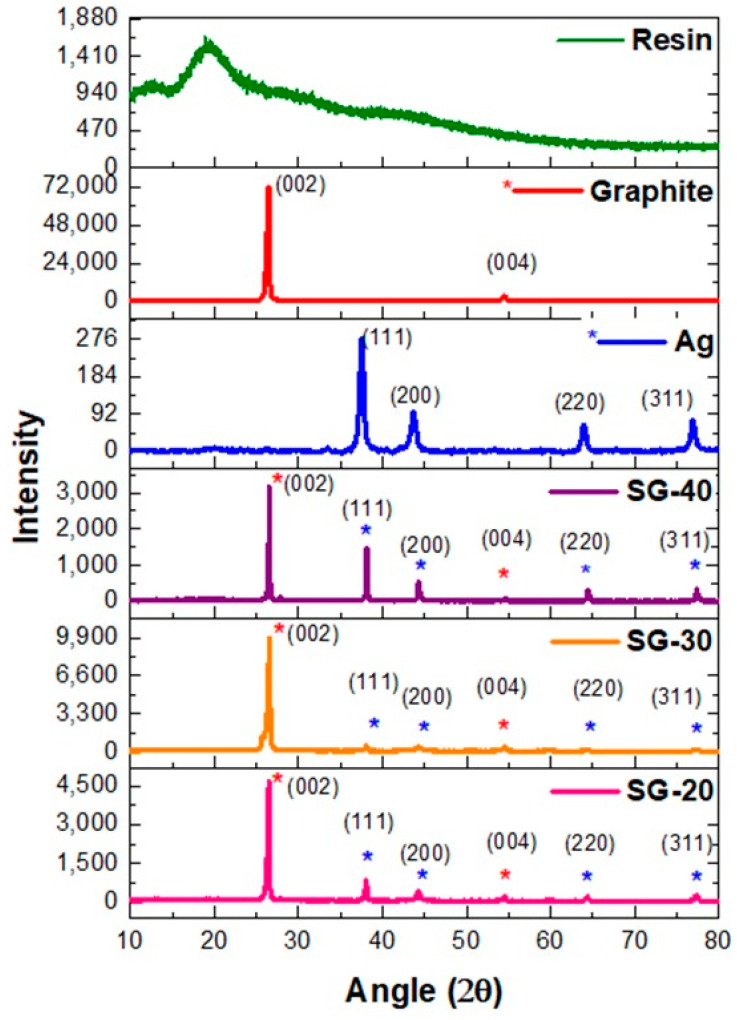
XRD results of resin, graphite, Ag, and graphite-Ag-resin composites.

Pure silver and its nanocomposites were characterized via XRD (Figure 3). Ag nanoparticles have shown four intense and sharp diffraction peaks at 38.1°, 44.4°, 64.4°, and 77.4°, reflecting 2.4 Å(111), 2.0 Å(200), 1.4 Å(220), and 1.2 Å(311) d-spacing (planes), respectively, which is in agreement with the results reported by Agasti [34] as well as perfectly matching with JCPDS card no. 03–0931. Meanwhile, nanocomposites of Ag-resin have exhibited the same diffraction pattern with similar d-spacing values and diffraction planes. Figure 4 has exhibited 2θ and d-spacing values for the S-20 nanocomposite at 37.7° d_(111)_ = 2.4 Å, 44.0° d_(200)_ = 2.1 Å, 64.0° d_(220)_ = 1.5 Å, and 77.1° d_(311)_ = 1.2 Å. Meanwhile, 2θ values were found at 38.1°, 44.0°, 65.1°, 77.1° and 38.1°, 44.1°, 64.2°, 77.1° for S-40 and S-60, respectively, having similar diffraction planes and d-spacing values to of pure Ag nanoparticles. These results declare that even after the mixing of silver nanoparticles in the resin, no silver oxide such as Ag_2_O is produced, thus confirming the successful blending and formation of this Ag-PFR nanocomposite.

XRD spectra of ternary graphite-Ag-resin nanocomposites have revealed the successful synthesis of composites (Figure 4). For the SG-20 composite, 2θ and d-spacing values were found at 26.4° d_(002)_ = 3.4 Å, 38.0° d_(111)_ = 2.4 Å, 44.2° d_(200)_ = 2.0 Å, 54.5° d_(004)_ = 1.7 Å, 64.3° d_(220)_ = 1.4 Å, and 77.3° d_(311)_ = 1.2 Å, while the SG-40 composite has almost similar results of 26.4° d_(002)_ = 3.4 Å, 38.0° d_(111)_ = 2.4 Å, 44.2° d_(200)_ = 2.0 Å, 54.5° d_(004)_ = 1.7 Å, 64.2° d_(220)_ = 1.5 Å, and 77.3° d_(311)_ = 1.2 Å. The composite SG-60 has shown diffraction signals at 26.4° d_(002)_ = 3.4 Å, 38.0° d_(111)_ = 2.4 Å, 44.2° d_(200)_ = 2.0 Å, 54.4° d_(004)_ = 1.7 Å, 64.3° d_(220)_ = 1.4 Å, and 77.3° d_(311)_ = 1.2 Å. These results (in Figure 4) have shown that there is no shifting of peaks, thus confirming the absence of oxide formation in any of the composites, even after the mixing of resin containing nanoparticles in situ.

### 4.3. Scanning Electron Microscopy (SEM)

**Figure 5:**
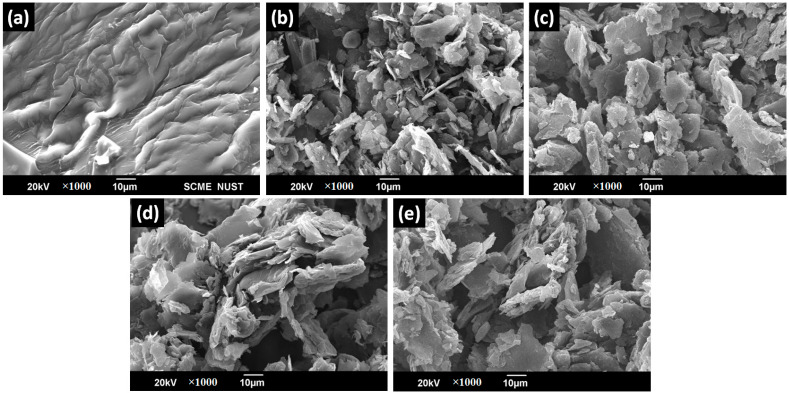
SEM micrograph of: (**a**) pure resin, (**b**) graphite, (**c**) G-20, (**d**) G-40, (**e**) G-60.

In Figure 5a, SEM micrographs have revealed the surface of resin. Previous studies [31] suggest that this might be due to the complex 3D polymerization between phenol and furfural. Such a high degree of polymerization has also ensured that there is no unreacted furfural or phenol left in the resin, resulting in the impartment of thermosetting behaviour along with crosslinking properties. Although it needs further clarification, similar reasons were assumed in the literature by Zeeshan et al. [31]. Figure 5b shows an SEM micrograph of graphite flakes in thin plate shapes which are randomly distributed over the entire area. A similar shape was reported in the literature [35]. XRD and EDS have also confirmed the purity of graphite. Figure 5c–e depicts SEM results of graphite–resin composites as smooth and homogenous. Micrographs have indicated graphite dispersion on the resin surface. As the graphite loading level increases, the lighter region also increases, confirming the conductive nature of graphite powder that was enhanced due to gold sputtering [31], while the non-conductive resin appears as the darker region. Due to the adhesive nature of resin, graphite powder is well merged in the composite.

**Figure 6:**
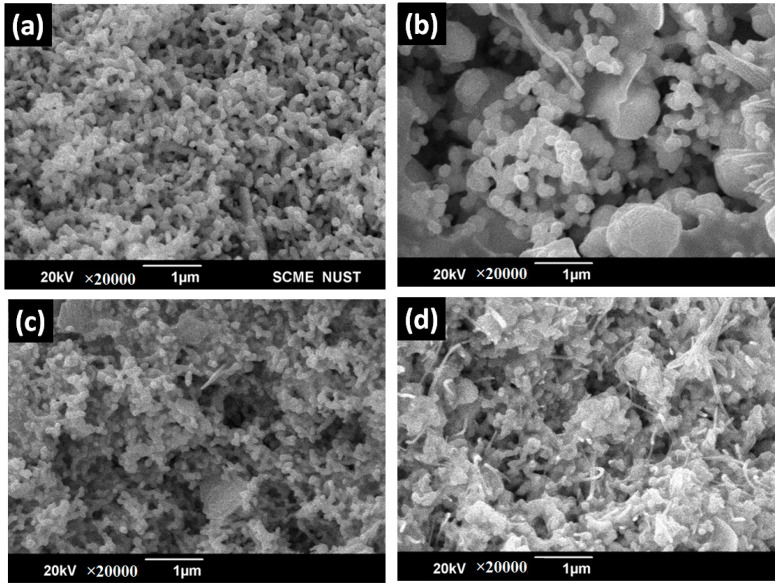
SEM analysis of Ag resin nanocomposites: (**a**) Ag NPs, (**b**) S-20, (**c**) S-40, and (**d**) S-60.

Figure 6a shows silver nanoparticle SEM micrographs. These nanoparticles were fused, spherical in shape, and had an average size of about 82 nm. Such findings match well with a surface analysis study in the reported literature [32]. The silver nanocomposite surface analysis in Figure 6b–d also indicates that spherical fused nanoparticles were well merged in the resins. The SEM images also confirmed that as the loading level of silver NPs increases from S-20 to S-60, the surface becomes denser with spherically shaped nanoparticles. These interconnected Ag nanoparticles caused an enhanced current to flow from one direction to the other, resulting in the formation of more electrically conductive silver nanocomposites. The saturation point arrived when the nanoparticles were added beyond 60 *w*/*w*% due to difficulty in mechanical stirring and agglomeration.

**Figure 7:**
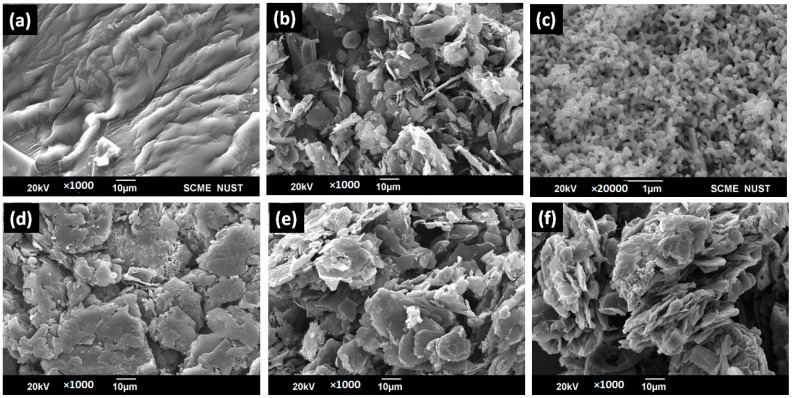
SEM results: (**a**) resin, (**b**) silver nanoparticles, (**c**) graphite, (**d**) SG-20, (**e**) SG-30, and (**f**) SG-40.

The micrographs of all the pure precursors including pure PFR, graphite, and Ag (shown in Figure 7a–c) along with surface analysis of ternary composites composed of both conductive nanoparticles blended with different weight percentages into resin have been studied and shown in Figure 7d–f. Since, these hybrid composites have both Ag NPs as well as graphite particles along with resin, therefore, larger aggregates become visible. Nano-structured silver particles were perfectly blended with the resin. All the silver–graphite composites exhibited a complex 3D surface, which is due to the branching and polymerization of the resin after curing.

**Figure 8:**
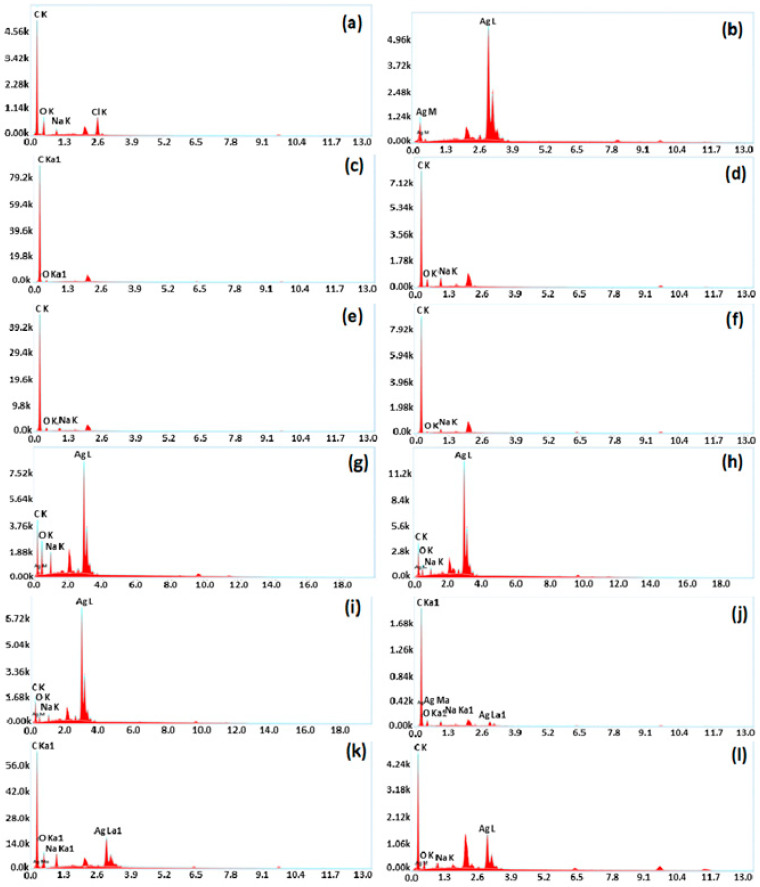
EDS results of (**a**) pure resin, (**b**) Ag NPs, (**c**) graphite, (**d**) G-20, € G-40, (**f**) G-60, (**g**) S-20, (**h**) S-40, (**i**) S-60, (**j**) SG-20, (**k**) SG-30, and (**l**) SG-40.

The elemental analysis of pure PFR is illustrated using energy dispersive X-ray spectroscopy with the gold sputtering, shown in Figure 8a. Carbon appeared as the highest moiety, while oxygen was detected as the second major element. Both are backbone elements of resin. Sodium was also detected because NaOH was used in the catalyst mixture. Pure Ag was detected as the single element in the elemental analysis, as shown in Figure 8b. The elemental composition of graphite powder is also depicted in Figure 8c. The presence of 4.7 *w*/*w*% oxygen can be attributed to oxygen functionalities at the surface. Figure 8d–f shows the EDS results of all the graphite composites G-20, G-40, and G-60, respectively, which only contained C and O as the major elements while sodium was used as the catalyst. The elemental composition of silver nanocomposites is also shown in Figure 8. The successful preparation of all the composites was confirmed by the increase in the weight% of silver content, which is represented in Figure 8g–i as 25.4 *w*/*w*%, 45.4 *w*/*w*%, and 54.1 *w*/*w*% for S-20, S-40, and S-60, respectively. The graphite–silver composite is shown in Figure 8j–l, which verifies the successful blending of silver with graphite and PFR. Elemental contents were found in agreement with the proportion of components (PFR-graphite-Ag) employed in composite formation.

### 4.4. Thermogravimetric Analysis

**Figure 9:**
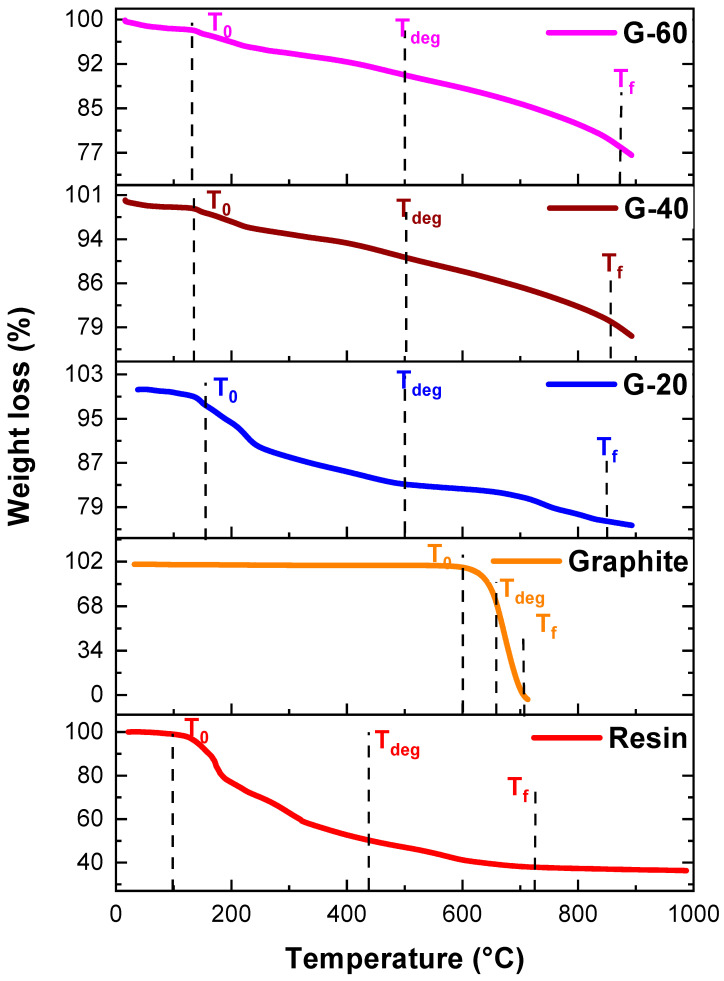
TGA analysis of resin, graphite, and graphite resin composite.

Thermal stability behaviour was checked for all pure resin, nanoparticles, and nanocomposites via TGA. For pure resin, onset temperature T_0_ (where decomposition started) was 96 °C, while decomposition temperature T_f_ (where decomposition ended) was 727 °C. T_deg_ was 438 °C, the temperature at which maximum degradation was achieved. Likewise, the TGA values of pure graphite were 600 °C and 705 °C for T_0_ and T_f_, respectively. Its thermal stability range was 26 °C to 600 °C. Degradation temperature increased from 438 °C to about 500 °C as the graphite powder was added. Detailed TGA results of binary composites of resin and graphite are described in Table 1 and Figure 9. The temperature of up to about 96 °C could also be related to the loss of water molecules, oligomers, and small residual monomers. Meanwhile, the temperature region from 96 °C to 727 °C results in overall degradation, in which chains break apart and different reactions regarding cross-linking occurred. Therefore, those structures which were previously formed partially decompose, resulting in the formation of carbonaceous residue beyond the temperature of 727 °C. Similar trends were observed by Zeeshan et al. [31].

**Figure 10:**
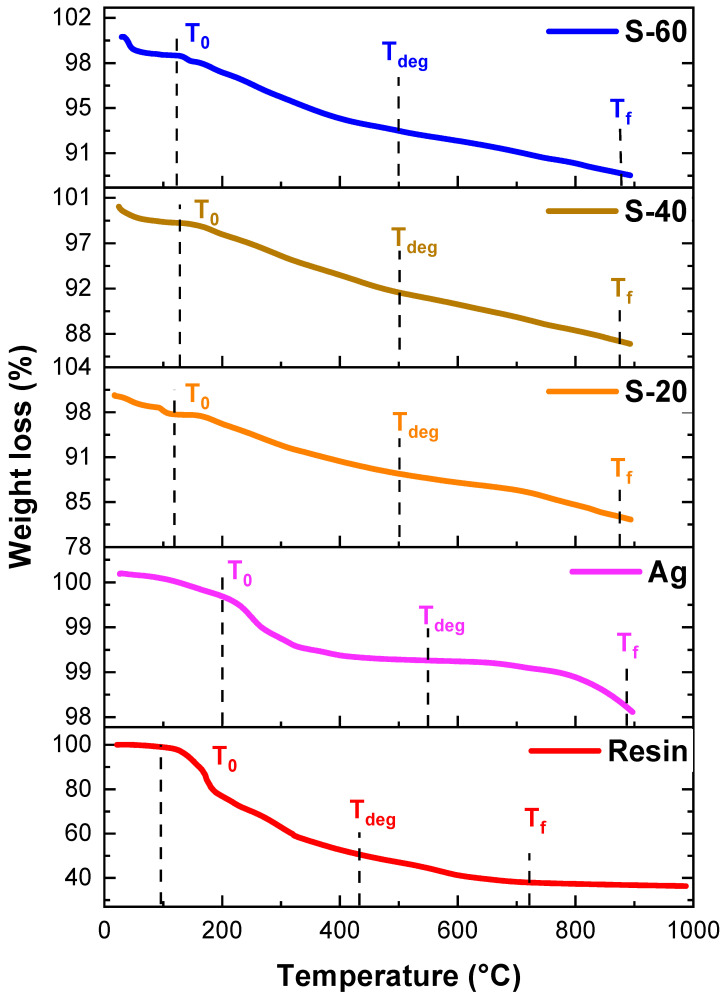
TGA graphs of resin, Ag, and silver resin nanocomposite.

Figure 10 exhibits TGA curves for silver nanoparticles and their binary resin composites. Temperatures T_f_, T_0_, and T_deg_ were 883 °C, 200 °C, and 549 °C, respectively, for silver nanoparticles. After blending with resin, their thermal stability was rechecked. It was observed that T_0_ values increased from 96 °C to 117 °C, 127 °C, and 122 °C for S-20, S-40, and S-60, respectively, because of silver nanoparticles which have increased thermal stability. Similarly, T_deg_ values also increased from 438 °C to 499 °C, from resin to S-60, confirming thermal stability increases due to the incorporation of nanoparticles (Table 1).

**Figure 11:**
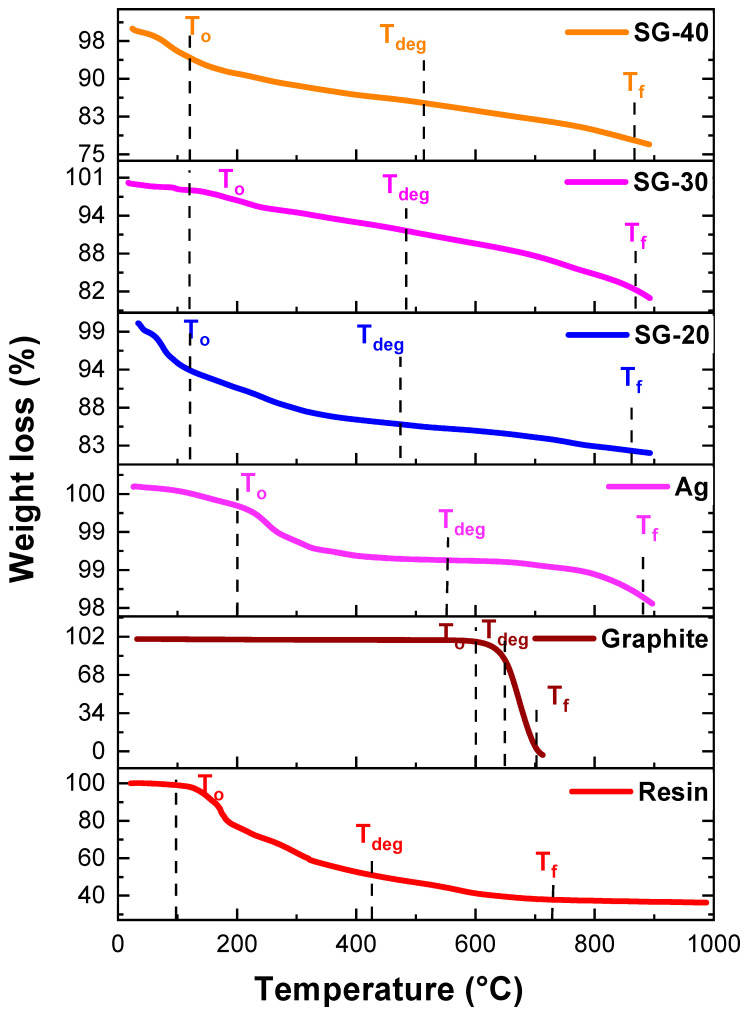
TGA graphs for Graphite/Ag/resin nanocomposites.

Figure 11 exhibits TGA curves for silver nanoparticles, graphite, and their ternary resin composites. It was observed that T_0_ values of resin increased from 96 °C to up to 120 °C for all ternary resin composites., whereas T_deg_ values increased from 438 °C to 474 °C, 484 °C, and 512 °C for S-20, S-40, and S-60, respectively (Table 1, Figure 11). These results have shown that upon incorporation of silver and graphite nanoparticles, there is a significant increase in thermal stability of ternary composites. These results also match with conventional phenolic resins [36]. Thermogravimetric results exposed that the introduction of graphite and metallic nanoparticles into the resin lowered the degradation rate, which could be related to the increase in crosslinking between nanoparticles and resin.

### 4.5. UV-DRS

**Figure 12:**
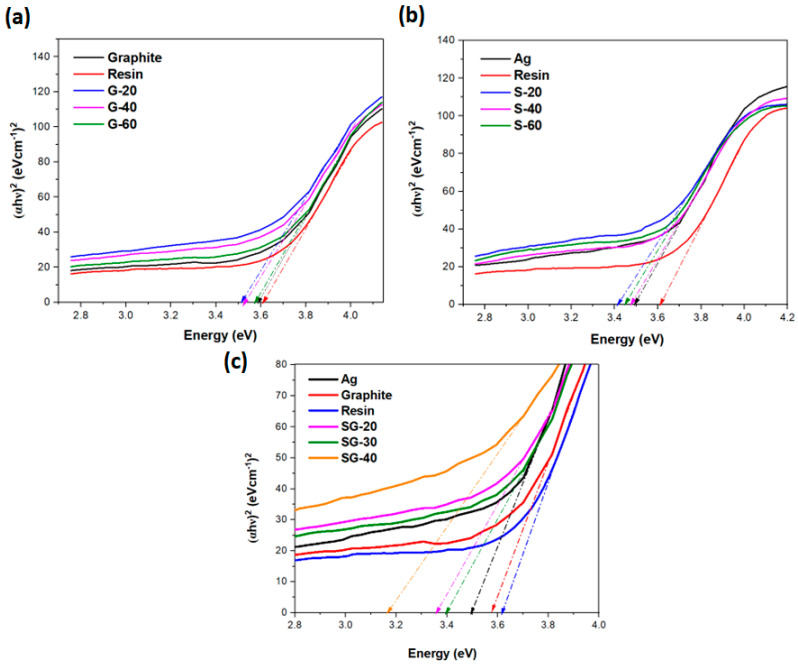
Tauc plot of (**a**) graphite-resin composites, (**b**) Ag-resin nanocomposites, and (**c**) graphite-Ag-resin composite.

A diffuse reflectance spectroscopic technique was used for the measurement of electrical conductivity. Band gap calculations were performed using Tauc plot calculations. Pure graphite extracted from dry battery cells has exhibited a bandgap energy value of about 3.5 eV. A similar value was reported by other researchers in the literature [37], while resin exhibited a higher bandgap energy value of about 3.6 eV. All the graphite–resin composites have shown bandgap energy values of slightly less than 3.5 eV. Thus, all graphite–resin composites are semi-conductors (Figure 12a).

Pure silver nanoparticles have exhibited a bandgap energy value of about 3.5 eV. A similar result was reported by AJ Das et al. [38]. All the silver–resin composites have shown bandgap energy values of less than 3.5 eV. Thus, all silver–resin composites are semi-conductors (Figure 12b).

The lowest bandgap energy value, i.e., 3.1 eV, was exhibited by the SG-40 (Figure 12c) Ag-graphite-resin nanocomposite, while the bandgap values for both nanocomposites SG-20 and SG-30 were about 3.3 eV, which is slightly less compared to the silver bandgap energy value. The lowest bandgap energy value of SG-40 could be due to the reason that silver contains one valance electron in the electronic structure as well as the optimized 20 *w*/*w*% of graphite loading that has synergistically performed for electronic conduction, while 40 *w*/*w*% of resin aided perfectly in providing adhesive, fine, and uncracked cured textured samples. SG-40 has exhibited the lowest bandgap because of good conductive path formation due to the synergistic effects of graphite and Ag filler particles when incorporated with PFR. These nanocomposites with a lower bandgap, better thermal stability, and increased electrical conductivity have the desired properties for use in parts of electronic applications and can be applied as conductive ink due to the lower bandgap energy value. Therefore, this lowered bandgap energy value and best electrical conductivity have the potential to be applicable at an industrial level.

### 4.6. Four Probe Technique

**Figure 13:**
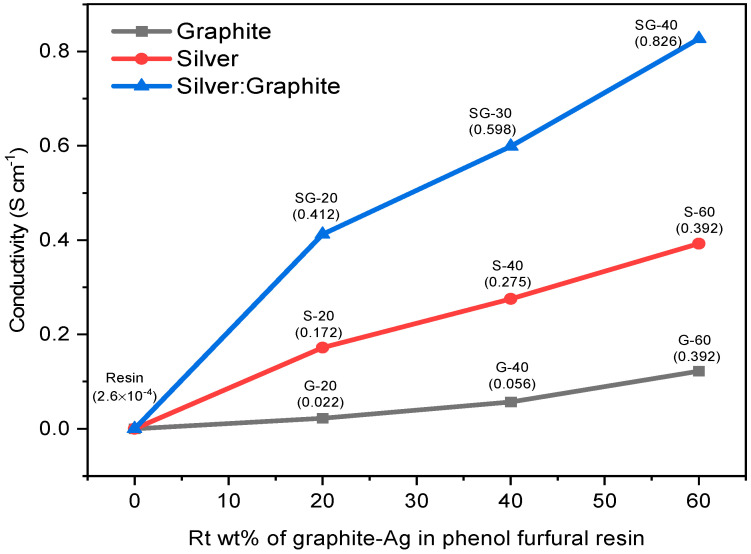
Electrical conductivity results of binary and ternary composites.

The electrical conductivity of the composites was indirectly measured using a four-probe method. Two probes at adjacent corners transfer a constant supply of current, while the remaining probes measure the difference in voltage between the two corners. Figure 13 shows the electrical conductivity results of graphite-Ag-resin binary and ternary composites. Pure resin has exhibited poor conductivity of the order 2.6 × 10^−4^ Scm^−1^, but conductivity significantly increased up to 2.2 × 10^−2^ Scm^−1^ by 20 *w*/*w*% of graphite–resin composite. This value of electrical conductivity was further increased to about 5.6 × 10^−2^ Scm^−1^ and 3.92 × 10^−1^ Scm^−1^ with 40 *w*/*w*% and 60 *w*/*w*% of graphite resin composite, respectively. Upon further addition of graphite, mechanical stirring is hindered, resulting in self-agglomeration and lump formation due to voids and spaces in the graphite powder.

To study the electrical properties of resin, silver nanoparticle metallic filler was also loaded in different percentages. The conductivity of Ag-resin nanocomposite film was calculated as 1.72 × 10^−1^ Scm^−1^, 2.75 × 10^−1^ Scm^−1^, and 3.92 × 10^−1^ Scm^−1^ upon addition of 20 *w*/*w*%, 40 *w*/*w*%, and 60 *w*/*w*% of Ag nanoparticles, respectively, which is far greater than the conductivity results of Ag NPs in resin of 2.48 × 10^−2^ Sm^−1^ reported by Ibrahim et al. [17]. These results have exhibited good electrical conductivities for Ag-resin nanocomposite film compared to graphite–resin composite because Ag has lower ionization energy at 7.57 eV while that of carbon is 11.26 eV. Therefore, Ag provides a rapid flow of electrons simply through applying less energy. Likewise, the highest conductivity was observed for SG-40 at about 8.26 × 10^−1^ Scm^−1^ as it consists of both silver and graphite nanoparticles in optimum concentration, while 4.12 × 10^−1^ Scm^−1^ and 5.98 × 10^−1^ Scm^−1^ were calculated for SG-20 and SG-30, respectively. The optimized composite SG-40 comprising 40 *w*/*w*% Ag and 20 *w*/*w*% of graphite furnished the best conductivity results.

This four-point probe technique also confirms that there exists a direct relationship between the added content of conducting nanoparticles up to a certain point, along with the decrease in bandgap energy values, and increased thermal conductivity in all the resin composites. Such nanocomposites with increased electrical conductivity, better thermal stability, and decreased bandgap energy value have the ability to be applied in numerous electronic parts and as conductive ink. Therefore, such composites have the potential to be applicable at the industrial level. However, further enrichment causes nonhomogeneous mixing as well as difficulty in mechanical stirring. It is proposed that both silver and graphite nanoparticles act as a junction in between polymeric chains of resin.

## 5. Conclusions

A renewable, native, and economical material, i.e., bagasse, was thrivingly used to extract furfural and then employed in green resin synthesis which served as the adhesive material between conductive fillers and the surface. Three distinct ratios of conductive fillers were loaded in the polymerized matrix to obtain the optimized adhesion and electrical performance of films. Various hybrids of nanocomposites were corroborated by the results of TGA, SEM, XRD, FTIR, four-probe technique, and EDS. Results showed a significant increase in the conductivity of pure resin and that of nanocomposites; an abrupt increase was observed when both nanoparticles were added. TGA, four-probe, and bandgap results have shown that PFR synthesized from bagasse can be loaded with conductive nanoparticles. Therefore, it is proposed that graphite and silver nanoparticles may act as the conducting junctions in between the different polymeric chains of resin, which ultimately increases thermal and electrical conductivity. So, it has good potential to be employed as an electrically conductive adhesive.

## Figures and Tables

**Table 1:** TGA results of silver–resin nanocomposites.

Pure Resin and Nanocomposites	Temperature (°C)		
	T_0_	T_deg_	T_f_
Bagasse resin	96	438	727
Ag	200	545	883
Graphite	600	658	705
G-20	154	499	847
G-40	133	500	855
G-60	130	499	874
S-20	117	499	874
S-40	127	500	871
S-60	122	500	876
SG-20	120	474	860
SG-30	120	484	866
SG-40	120	512	865

## Data Availability

The data that support the findings of this study are available on request from the corresponding author.
